# Teaching in hunter–gatherer infancy

**DOI:** 10.1098/rsos.150403

**Published:** 2016-01-20

**Authors:** Barry S. Hewlett, Casey J. Roulette

**Affiliations:** Department of Anthropology, Washington State University, Vancouver, WA, USA

**Keywords:** teaching, hunter–gatherers, social learning

## Abstract

A debate exists as to whether teaching is part of human nature and central to understanding culture or whether it is a recent invention of Western, Educated, Industrial, Rich, Democratic cultures. Some social–cultural anthropologists and cultural psychologists indicate teaching is rare in small-scale cultures while cognitive psychologists and evolutionary biologists indicate it is universal and key to understanding human culture. This study addresses the following questions: Does teaching of infants exist in hunter–gatherers? If teaching occurs in infancy, what skills or knowledge is transmitted by this process, how often does it occur and who is teaching? The study focuses on late infancy because cognitive psychologists indicate that one form of teaching, called natural pedagogy, emerges at this age. Videotapes of Aka hunter–gatherer infants were used to evaluate whether or not teaching exists among Aka hunter–gatherers of central Africa. The study finds evidence of multiple forms of teaching, including natural pedagogy, that are used to enhance learning of a variety of skills and knowledge.

## 1. Introduction

Social–cultural anthropologists, cognitive psychologists, educators and evolutionary biologists are interested in teaching, but the different disciplines have distinct histories and research traditions that have influenced the characterizations and representations of teaching in humans (see [1,2] for critiques). As detailed further below, anthropologists conducting research in small-scale cultures around the world often indicate it is rare or non-existent, cognitive psychologists and some educators indicate it is unique to humans and part of human nature, while evolutionary biologists suggest it exists in several non-human animal species and that it does not require complex cognitive abilities. This paper briefly reviews the strengths and weaknesses of these diverse disciplinary perspectives and then examines whether or not teaching exists among Aka hunter–gatherers, one of the last active forager groups on the earth.

It is important to understand whether or not teaching exists cross-culturally because some have suggested that teaching is one of three key cognitive abilities that enabled humans to develop cumulative culture and adapt to environments all around the world [3,4]. Teaching, language and accurate imitation are a triumvirate that may contribute to the high fidelity of the transmission of cultural beliefs and practices in humans [5]. When cultural elements are highly conserved they remain in the population longer, which increases the chance of an innovation or modification of a cultural belief or practice, which in turn leads to more elaborate cumulative culture [6]. Expansions in cumulative culture amplify the number of socially transmitted beliefs and practices, which may ultimately lead to an increasing importance of teaching. Simulation models indicate that transmission fidelity (measured by the loss rate of a belief or practice) is far more important than innovation to establishing and maintaining cumulative culture [7]. The studies cited in this paragraph indicate that teaching is a key feature of humanity that enabled pronounced cultural diversity.

### 1.1 The teaching debate

#### 1.1.1 Teaching does not exist or is rare in small-scale cultures

For decades, social–cultural anthropologists and cultural psychologists have been interested in identifying and explaining the differences in how children learn in ‘primitive’ versus ‘civilized’, Western versus non-Western, formal versus informal and WEIRD (Western, Educated, Industrial, Rich, Democratic [8]) versus non-WEIRD cultures. Researchers have had a long history of interest in these dichotomous comparisons [9] in part because researchers sought to illustrate the biases in Western educational systems.

Mead [10] was one of the first social–cultural anthropologists to identify differences between ‘primitive’ and ‘civilized’ educational systems. Mead [11] characterized small-scale societies as ‘learning cultures’ because the learners initiated the acquisition of skills or knowledge, culture was acquired relatively easily, and everyone agreed upon what was important to learn. By contrast, ‘civilized’ societies were characterized as ‘teaching cultures’ because the teachers decided what was important to transmit to the young. Mead indicated that Western teaching and educational systems emerged from Western institutions that were interested in conversion, indoctrination and colonization.

Since the 1970s, social–cultural anthropologists and cultural psychologists [12–14] continued the tradition of comparing learning in ‘informal’ small-scale cultures and ‘formal’ politically and economically stratified cultures. The small-scale versus stratified culture comparisons maintained the impression that Western-style teaching was rare in small-scale cultures.

Some social–cultural anthropologists have become particularly adamant that teaching does not exist in small-scale cultures [15]. Lancy's review article [16] ‘Learning “from nobody”: the limited role of teaching in folk models of children's development’ concludes that ‘Teaching—even if defined, minimally, as self-conscious demonstration—is rare in the accounts of anthropologists and historians… Teaching has largely been superfluous in the process of cultural transmission throughout human history’ [16], p. 97. Lancy uses the term teaching to refer to ‘student-centered, developmentally appropriate instruction by dedicated adults’ [16] associated with Western schools. Rogoff [17] also minimizes the importance of teaching in an article ‘Childhood and learning: how do children learn without being taught? One way is by observing and pitching in’. Like Mead and the others that followed, she compares learning processes in a Mayan community with learning in Western schools. Western schools are characterized by assembly-line instruction where teachers manage what is learned and transmit knowledge verbally, whereas learning in the Mayan community emphasizes intent participation where children learn by observing, listening in and participating in adult activities [14].

Not all social–cultural anthropologists agree with the researchers mentioned above. Kruger & Tomasello [18] and Garfield *et al.* [19] find multiple examples of teaching in their cross-cultural surveys of learning in humans, and Kline *et al.* [20] provide examples of teaching in Fijian villages. In terms of the Aka in this study, Hewlett *et al.* [1] and B. L. Hewlett [21] provide several anecdotes of teaching. The results of these anthropological studies are different from those described above, because the researchers used definitions of teaching from cognitive psychology or evolutionary biology discussed below.

Social–cultural anthropologists have contributed significantly to our understanding of learning, because their research demonstrates that children can learn complex skills and knowledge very quickly, easily and early in life without Western-based curricula and approaches to education [22]. Several of the ‘informal’ ways of learning, such as situated learning [13] and guided participation [23], have been incorporated into contemporary Western classrooms. Their research has contributed to our understanding of learning, but has not contributed much to our understanding of teaching in part because of their Western-centric conceptions of teaching.

#### 1.1.2 Teaching is part of human nature and unique to humans

This is the position of several cognitive psychologists [5,24] and some educators [25]. Tomasello *et al*. [5] indicate that humans have the abilities to read the intentions of others, view others as intentional agents, and internalize adult's behaviour or instructions (i.e. metacognition), and that these cognitive abilities contribute to efficient scaffolding and teaching in humans. They define teaching as ‘a behavior in which one animal intends that another learn some skill or acquire some bit of information or knowledge that it did not have previously’ [18], p. 374. According to these authors, adults' expectation that children will learn and the adults' intent to provide assistance if and when the need arises is what distinguishes humans from other primate species. Instructed learning (another term for teaching) emerges at age 4, the age at which children are able to attribute false beliefs, i.e. the cognitive ability to recognize that others have beliefs that are different from one's own [18].

Strauss & Ziv [25] summarized research on the development of children's teaching, found multiple instances of teaching occurring outside of school contexts, and hypothesize that teaching is a natural human capacity. In other studies, they discovered that children as young as three demonstrated games to other children to help them learn how to play [26]. Teaching is defined as ‘an intentional activity that is pursued in order to increase the knowledge (or understanding) of another who lacks knowledge, has partial knowledge or possesses a false belief’ [25], p. 187. Theory of mind (i.e. attribution of false beliefs) is a key component of their definition, which means that by comparison to other primates, teaching is unique to humans.

Gergely & Csibra [24] take a slightly different view of teaching. Teaching does not require a theory of mind or intent. They describe one form of teaching, called natural pedagogy, which they hypothesize is an innate and relatively unique feature of human cognition. Natural pedagogy involves an individual (the teacher) providing explicit signals (e.g. pointing, motherese, child-directed speech, infant's name or eye gaze) of generalizable (to other situations or individuals) knowledge to another individual (the learner) who can read and interpret the content of the signals [24], p. 5. They hypothesize that natural pedagogy evolved to solve the recurring problem of faithfully transmitting opaque knowledge about tools (e.g. about their functions) to a learner. Learners evolved to pay attention to these ‘ostensive’ cues and teachers evolved the skills to convey important information to learners by using these cues. Laboratory experiments find that infants are more likely to imitate and learn about novel objects when caregivers use ostensive cues. The researchers indicate that the learning processes emphasized by social–cultural anthropologists, such as participation, observation and imitation, are not sufficient for learning tasks and knowledge that are opaque to the learner. However, Csibra & Gergely [27] concede that learning is more effective when learners trust teachers so that reading the intentions of others facilitates, but is not necessary for natural pedagogy.

The systematic and detailed laboratory research methods of cognitive scientists have contributed important insights into understanding the relatively unique genetically based features of human cognition. It would be difficult for social–cultural anthropologists who often use more qualitative and open-ended methods to identify these features of human cognition, but social–cultural anthropologists are critical of these approaches because they rely primarily on studies in laboratories with children from WEIRD cultures. The natural pedagogy hypothesis is based on laboratory studies of parents and infants from WEIRD cultures, where infant directed speech, motherese, face-to-face interaction and formal teaching are common, so it is not surprising from a cultural anthropologist's perspective that such studies find evidence of natural pedagogy.

#### 1.1.3 Teaching exists in humans and other non-human animals

The approaches described above generally require the learner and teacher to have particular cognitive mechanisms for teaching to occur—intent, reading the intentions of others (theory of mind) or metacognition—and generally conclude that non-human animals do not have teaching. By contrast, evolutionary biologists indicate that teaching exists in several species. They have been interested in social learning in non-human animals for a long time [28], but it was not until 1992, when Caro & Hauser [29] provided an overview and operational definition of teaching, that systematic research began to accumulate. Their definition indicates that teaching exists when a knowledgeable individual modifies its behaviour only in the presence of a naive individual, the knowledgeable individual incurs some cost or derives no immediate benefits by modifying its behaviour, and the naive individual acquires knowledge or skills more rapidly or efficiently than it would otherwise, or that it would not have learned at all as a result of the individual's behaviour.

The Caro and Hauser definition enabled animal behaviour researchers to systematically evaluate whether teaching existed in non-human species. Current research indicates teaching exists in meerkats, ants and babblers and that it may occur in several other species, such as cheetahs [30]. For instance, meerkat adults modify their behaviours (e.g. provide dead, then disabled with the stinger removed and then live scorpions to juveniles) to help juveniles learn how to handle and kill them for consumption. The adults modify their behaviour according to the purring sounds (an indicator of the juvenile's age) of the pup.

### 1.2 Overview and working definition

The various approaches offer their strengths and weaknesses. Social–cultural anthropologists have extensive field studies of learning in diverse cultures; cognitive psychologists use clear and precise methods to control for multiple factors; and evolutionary biologists provide a cross-species perspective that can help us understand the selective pressures that lead to the evolution of teaching.

Each disciplinary approach also has biases and limitations. Social–cultural anthropologists and cultural psychologists use Western-centric conceptions of teaching, cognitive psychologists use anthropocentric definitions and conduct their studies with WEIRD children, and evolutionary biologists use an animal-centric definition that focuses on behaviour at the expense of neglecting underlying cognitive structures.

We prefer to start with a minimal definition of teaching: an individual modifies her/his behaviour to enhance learning in another (see [1,2,31] for similar definitions). Teaching cannot be a by-product of another activity. For instance, if someone in a forager camp sees a poisonous snake and yells ‘snake’ this may provide a learning opportunity for others but it is not teaching because the individual did not modify his or her behaviour to enhance the learning in another; the learning is a by-product of warning others about the snake. We also agree with Thornton & Raihani [30] that contingent communication or some sensitivity between teacher and learner, which they call ‘behavioural coordination’, is an important feature of teaching.

### 1.3 Hypotheses

The primary aim of this study is to evaluate whether or not any form of teaching, as defined above, exists in foragers. We focus on infancy in particular to determine whether or not the type of teaching described by Gergely & Csibra [24], i.e. natural pedagogy, occurs in hunter–gatherer infancy. We concentrate on the teacher's rather than the learner's side of natural pedagogy, i.e. the teacher's use of ostensive cues such as pointing or calling the infant's name to draw the infant's attention to opaque knowledge or skills. Our study is consistent with Strauss & Ziv's [25] emphasis on trying to understand teacher's natural cognitive abilities. Some anthropologists and evolutionary biologists have made other predictions about how and when teaching should occur and we use our limited data to evaluate the following additional hypotheses.

(1) Teaching by parents should occur as early in life as possible rather than later so as to free up a parent's time to invest in their next child [32].
(2) Teachers are likely to be parents [32] or others who are biologically related to the learners [19,32] due to the costs of teaching.
(3) Teaching should be limited to skills difficult to learn and domains of life highly valued and necessary for success in a culture [20,33].

### 1.4 Aka hunter–gatherers

The Aka are one of about 15 ethnolinguistic groups of Congo Basin hunter–gatherers [34]. About 40 000 Aka live in northern Republic of the Congo and southern Central African Republic and about 2000 live in and around the study area. The Aka live in mobile groups of 25–35 people and rely upon a wide variety of hunting and gathering techniques for day-to-day subsistence. The Aka have multidimensional social–economic relationships with Ngandu and other farming ethnic groups. As with several forager groups, three related foundational schema—ways of thinking that pervade many domains of life—include egalitarian ethos, autonomy and sharing. An egalitarianism ethos devalues hierarchical ranking, including political, age or gender ranking. Men and women of all ages are viewed as relatively equal and have similar access to resources. Respect for individual autonomy is also a core value. One does not coerce or tell others what to do, including children. Giving or sharing is also a pervasive way of thinking in Aka life; they share 50–80% of what is acquired, they share it with everyone in camp, and they share it every day. Sharing of childcare is also extensive; for instance, 90% of Aka mothers report that other women nurse their young babies [1].

## 2. Methods and analysis

Ten (five males and five females) Aka 12–14-month-old infants were videotaped for 1 h in a naturalistic setting (usually in or near the camp). Infants came from nine different Aka camps within 3 km of the village. Caregivers and others in camp were asked to maintain normal activities as best as possible, but infants had to be awake and parents were asked to keep infants in public (not in hut) as a condition of Institutional Review Board approval, which was not difficult because families spend most of the day outside. Researchers have conducted infant focal follow observations for several years with Aka in the study area so filming was not that unusual for parents and community members. The video camera was set up in camp for about 30 min before filming started to help diminish attention paid to the camera by the infant and camp members. We wanted to return to the field to increase the sample size but a civil war in the Central African Republic made that impossible.

Hewlett made the videos and watched two of the videos carefully to establish a tentative coding scheme. Videos were sent to Gyorgy Gergely to verify whether the observations and coding of natural pedagogy were consistent with how it was evaluated in their laboratories. The coding system outlined in table 1 was established and both authors independently coded all tapes. The authors discussed discrepancies in coding and easily resolved differences. Finally, an individual unfamiliar with the hypotheses was trained with the codes and then coded two randomly selected videotapes. The outsider and researchers agreed on the coding of types of teaching to a satisfactory degree (Cohen's *κ*=0.734).

**Table 1.** Definitions of codes used in analysis of videotapes.

types of teaching coded	
	caregiver modifies his/her behaviour to enhance learning in infant. Must involve a skill (e.g. use of knife, how to hold baby) or knowledge (e.g. where to dig)
natural pedagogy	caregiver points, uses eye contact, child-directed speech, infant name, or other cues to draw the infant's attention and provide information about a skill (how to use knife, machete, digging stick, climb tree) or knowledge (e.g. how to share, where to find roots)
positive feedback	caregiver smiles, makes positive sounds (*eee*) or dances in response to infant's good performance of a skill
negative feedback	caregiver makes displeasing comments or sounds or moves infant's body when the infant slaps, threatens, hits another or starts to do something that may be dangerous (pointing knife at person, climbing tree)
redirect	caregiver redirects infant to another location or activity because he/she does something dangerous (going into fire, tipping hot pot on fire), or inappropriate (tries to step into mortar)
opportunity scaffolding	caregiver provides infant with an object (e.g. knife, machete, digging stick) and learning opportunity. Carer may watch/monitor after providing the object, but does not provide cues about how to use
demonstration	caregiver demonstrates how to do particular task (use knife, etc.). Object may be given to infant after demonstration; includes demonstration by moving infant's body
task assignment	caregiver gives infant task (e.g. to bring something across camp)
move body	caregiver moves infant's body to show her/him how to dance or what not to do (the movement must convey information)
verbal instruction	caregiver provides some verbal explanation (making sounds not enough) about a task or knowledge to the infant
distribution teaching	caregiver turns infant on his/her lap so the infant faces towards other members in the camp

**other behaviours coded**	
caregiver or infant points	caregiver or infant points to something even when it is not clear whom or what is being pointed to
imitation	caregiver or other does task and infant imitates right after observing the task
practice play	infant practices or plays with knife or other object without intervention of others
general play	infant engages in general play with objects (sticks, bushes, etc.) or people

Coding of teaching was limited to skills or knowledge. If an infant was playing with a stick or throwing a piece of fruit and the caregiver modified his/her behaviour to help the infant play it was not coded as teaching because the skill or knowledge being acquired was not clear. Carer pointing was not coded if it occurred in natural pedagogy because it was part of the definition, but it was coded in other contexts. For instance, if a caregiver was holding an infant and then pointed to something on the other side of the camp, pointing was coded but not considered a teaching event. If pointing occurred to draw the infant's attention to how to determine when a yam on the fire was ready to eat, it was coded as natural pedagogy. Coding of imitation was limited to instances when the infant observed somebody performing a skill and then imitated the action shortly thereafter. Verbal explanation was limited to caregivers providing some explanation about a particular skill or knowledge.

All types of teaching coded required some modification of the caregiver's behaviour to help the infant learn as well as some contingent sensitivity or coordination. Contingent sensitivity is a feature of natural pedagogy, but may not be obvious with other types of teaching. For instance with negative feedback, the caregiver must at least attend to the infant's behaviour (what are they doing wrong?) and the infant has to respond to the negative comments or body movements in some way.

Some may question the inclusion of ‘distribution teaching’. Caregivers modified their behaviour by turning infants in their lap to face other members of the camp. What they are learning is not clear because our methods (one camera focused on infant) did not allow us to capture interactions and potential learning from others. We included it as a type of teaching because it does not occur among higher primates and Ochs & Schieffelin [35] described this as an important method for infant language acquisition.

A teaching ‘event’ was an instance of teaching activity from those listed in table 1. A teaching ‘episode’ was defined as a sequence of teaching events, practice play or imitation, that focused on acquiring the same skill or knowledge and occurred without an interruption of more than 15 s. If nothing happened for more than 15 s, it was considered the end of an episode. If a type of teaching event occurred a few times within an episode (e.g. a caregiver demonstrates how to use a knife, waits 5 s and then demonstrates again), it was recorded as a teaching ‘event’ only once for analysis. For the purpose of calculating the length of a teaching episode, time spent in practice play and imitation were omitted. An episode could include one to several teaching events. The duration of four behaviours were recorded—distribution teaching (time infant was faced out on caregiver's lap), practice play (practising a particular skill), general play (general play not clearly linked to a skill or knowledge), the length of teaching episodes (without practice play and imitation). The relationship and age (mother, father, other adult, older (greater than 5 years) child and similar-aged child) of the learner to the teacher was also recorded for each teaching behaviour.

The observational method is called ‘naturalistic’ because it took place in the camp during regular daily activities. Most of the taping took place during morning and late afternoon hours before people went out or just returned from the fields or the forest. This is a relaxing time when people often sit around and talk, visit and prepare and eat meals. Consequently, the timing of taping probably influenced some infant–caregiver interactions because caregivers were not working or engaged in subsistence activity during the taping, i.e. they had more leisure time with their infants. As with any group of individuals, some infants were outgoing, crawled in Hewlett's lap, and were not impacted by the camera. A few infants were very shy, not so sure about the camera and stayed away from its location.

## 3. Results

A total of 169 teaching events and 112 teaching episodes were observed during the 10.1 h of videotapes. Teaching episodes were relatively short. The mean episode lasted 20.3 s and for 47% of the episodes length was less than 3 s. The s.d. was 40.0 s and demonstrates enormous variability. Table 2 summarizes the frequencies of the different types of teaching observed in the videotapes. The means and standard deviations of the types of teaching are similar, which indicates pronounced intracultural variability. For instance, two infants did not experience natural pedagogy while one infant had 10 exposures to this type of teaching. The table indicates that infants received regular teaching episodes and that natural pedagogy, negative feedback, and demonstrations were the most common types of teaching.

**Table 2.** Frequency of different types of teaching events and related behaviours among Aka foragers.

type of teaching	percentage of videotapes with examples	mean frequency (s.d.) per hour videotape
natural pedagogy	80	4.1 (3.5)
positive feedback	50	1.2 (1.9)
negative feedback	60	2.7 (3.7)
opportunity scaffolding	50	0.9 (1.3)
demonstration	70	3.0 (3.2)
task assignment	60	1.4 (2.2)
move body	80	1.7 (1.5)
verbal instruction	40	0.6 (0.8)
redirect	50	1.3 (2.2)
teaching episodes	100	11.2 (5.0)

**other behaviours**		
imitation	70	2.5 (2.8)
caregiver points	100	12.4 (7.2)
infant points	60	1.8 (3.0)

Infants imitated someone about two or three times per hour (this includes imitation associated with a teaching event (68% of imitation cases) or simple observation of someone and then imitating). A surprising result was the high frequency (more than 12 times an hour on average) that caregivers pointed (not including pointing in natural pedagogy). Sometimes caregivers pointed during a teaching episode but 67% of the time caregivers pointed to people or objects in camp but it was not possible to evaluate precisely who/what was being pointing at because the film focused on the infant.

In terms of duration of particular behaviours, infants spent an average of 18.3 min (s.d.=14.0) on their caregiver's lap facing out towards the group, which was about 30% of the time the infants were observed. This is remarkably similar to the 31.1% of time Aka infants were observed in their caregiver's lap (usually facing out) with a much larger sample (20 infants), over a substantially longer period of time (9 h per infant), in more diverse contexts (e.g. camp and forest) [36]. Aka infants spent an average of 14.7 min (s.d.=13.0) in general play and 9.4 min (s.d.=9.1) in practice play.

The frequency and duration data also indicate that Aka teaching is often physical/proximal and seldom involves verbal explanation. Infants were on their caregiver's lap facing others 30% of the time and teaching by moving the infant's body occurred three times per hour (1.7 of move body plus 1.3 redirect where move body was part of the definition) while verbal explanation occurred only six times in the 10 h of videotapes.

Table 3 summarizes the various skills and knowledge that were transmitted with the different types of teaching. A remarkably diverse set of skills and knowledge were transmitted with the different forms of teaching. It is important to note that the skills and knowledge were transmitted by ‘teachers’ but we did not systematically measure, and therefore do not precisely know, what infants actually learned. We do know that with natural pedagogy and demonstration forms of teaching the infants imitated the teacher immediately after the teaching event about 40% of the time (see below).

**Table 3.** Skills and knowledge that were the focus of different types of teaching.

type of teaching	
natural pedagogy	how to use a knife to cut, how to dig for roots/yams, how to prepare food, how to build a fire, how to cook on a fire and how to hold a baby
positive feedback	how to dance or sing and how to train a dog
negative feedback	do not hit other children, do not take too much food, do not use machete to hit another, be careful not to tip pots when eating, be careful when holding a newborn
opportunity scaffolding	infant is given digging stick, knife, machete or axe while caregiver watches and monitors
demonstration	caregiver demonstrates how to use a machete or digging stick to search for roots/yams, how to dance and clap, how to put branch in ground to build house, how to chop with a knife
task assignment	infants given tasks to transport food, bowls, water bottle, knife and pots to others in camp
move body	caregiver moves infant's body to eat together, put food in pot, learn to dance
verbal instruction	how to dig roots, how to build a fire
redirect	Infant is moved or redirected to another area because she/he slaps another person, tries to hit someone with stick, uses a knife too close to others, almost falls out of a tree

Tables 4 and 5 examine the co-occurrence of different types of teaching and related behaviours. Table 4 lists the frequency at which various forms of teaching occur on their own or with other types of teaching in a particular teaching episode. For instance, in some cases a caregiver demonstrated a particular skill to an infant without pointing or verbal explanation (demonstration without co-occurrence with other forms of teaching) but at other times the caregiver may use positive feedback, body movement and demonstration to teach (co-occurrence of types of teaching) a skill. Most types of teaching occurred with other types, but negative feedback took place on its own about 50% of the time and pointing by the caregiver occurred independently (at least with this method) more than 70% of the time. Although caregiver pointing occurred independently, the link to teaching was not clear because the camera focused on infant interactions. Independent negative feedback events generally involved an infant hitting or pushing others and members of the camp responding by making irritated noises or redirecting (also with a higher percentage of independent events) the infant to another area.

**Table 4.** Frequency that teaching and related behaviours occur on own or with other forms of teaching and related behaviours.

type of teaching	total no. events	no. events that co-occurred in teaching episode	no. events that occurred on own
natural pedagogy	41	34	7
positive feedback	12	10	2
negative feedback	27	14	13
opportunity scaffolding	9	7	2
demonstration	30	26	4
task assignment	14	14	0
move body	17	15	2
verbal instruction	6	6	0
redirect	13	9	4

**other behaviours**			
imitation	25	17	8
caregiver points	124	34	90
infant points	18	13	5

**Table 5.** Proportion that different types of teaching, imitation and practice play co-occurred within a teaching episode. The total number of times a particular type of teaching co-occurred with another type of teaching is in parentheses in the first column. Co-occurrence rates are listed by row. For instance, natural pedagogy occurred 34 times in the 10 h of taping and it co-occurred with positive feedback 15% of the time, negative feedback 3% of the time, demonstration 59% of the time, etc. NP, natural pedagogy; PF, positive feedback; NP, negative feedback; OS, opportunity scaffolding; DEM, demonstration; TK, task assignment; RD, redirect; MB, move body; VERB, verbal explanation; POC, caregiver points; POI, infant points; I, imitation; PP, practice play.

	NP	PF	NF	OS	DEM	TK	RD	MB	VERB	POC	POI	I	PP
NP (34)		0.15	0.03	0	0.59	0.09	0	0.09	0.12	0	0	0.41	0.35
PF (10)	0.50		0	0	0.10	0	0.10	0.10	0.10	0	0	0.40	0.10
NF (14)	0.07	0		0.07	0	0	0.43	0.43	0.07	0.43	0	0	0
OS (7)	0	0	0.14		0	0	0	0.43	0	0.14	0	0	0.43
DEM (26)	0.77	0.04	0	0		0	0	0.11	0	0.04	0	0.42	0.23
TK (14)	0.21	0	0	0	0		0	0	0.07	0.71	0.21	0	0.07
RD (9)	0	0.11	0.67	0	0	0		0	0	0.33	0	0	0
MB (15)	0.20	0.07	0.40	0.20	0.20	0	0		0	0.13	0	0	0.07
VERB (6)	0.60	0.17	0.17	0	0	0.17	0	0		0.17	0	0.33	0.33
POC (34)	0	0	0.18	0.03	0.03	0.29	0.09	0.06	0.03		0.29	0.06	0.03
POI (13)	0	0	0	0	0	0.23	0	0	0	0.77		0	0
I (17)	0.82	0.23	0	0	0.65	0	0	0	0.12	0.12			0.29
PP (25)	0.48	0.04	0	0.12	0.24	0.04	0	0.04	0.08	0.04		0.20	

Table 5 summarizes the co-occurrence of specific types of teaching. Natural pedagogy often occurred with a demonstration and both of these types of teaching took place with imitation and practice play during a teaching episode. Task assignment often occurred when the parent pointed to a location where the infant should take the object or food. Opportunity scaffolding often took place with moving the infant's body, usually to place a knife or machete into her hand. Negative feedback and redirect also often co-occurred, generally in contexts described above for negative feedback. Positive feedback and verbal explanation rarely were observed but were most likely to co-occur with natural pedagogy. For instance, after a caregiver pointed to how to hold one's arms to carry a baby and then demonstrated how to hold the newborn, the infant might try to do it, after which the caregiver would smile or clap her hands. Infant pointing was also relatively infrequent but most likely to be observed during an episode when the caregiver pointed to somebody/something in camp. The table shows that they co-occur but it does not mean that teaching was taking place.

Table 6 considers from whom infants were learning. Parents were most likely to use a diverse array of types of teaching, in part because infants are held by or are in proximity to their mother or father much of the day [37]. Other adults or older children were most likely to use negative feedback or redirect to help infants learn what they can and cannot do (e.g. not to hit others or take their food).

**Table 6.** Proportion that different types of teaching occurred among different teacher–learner relationships. Total number of teaching events is in parentheses.

	mother	father	other adult(s)	older child	similar aged child
natural pedagogy (41)	0.70 (29)	0.12 (5)	0.10 (4)	0.05 (2)	0.02 (1)
positive feedback (12)	0.33 (4)	0.42 (5)	0.08 (1)	0.17 (2)	0.00 (0)
negative feedback (27)	0.33 (9)	0.00 (0)	0.52 (14)	0.15 (4)	0.00 (0)
opportunity scaffolding (9)	0.78 (7)	0.00 (0)	0.11 (1)	0.11 (1)	0.00 (0)
demonstration (30)	0.73 (22)	0.13 (4)	0.03 (1)	0.07 (2)	0.03 (1)
task assignment (14)	0.79 (11)	0.07 (1)	0.07 (1)	0.07 (1)	0.00 (0)
redirect (13)	0.23 (3)	0.00 (0)	0.23 (3)	0.54 (7)	0.00 (0)
move body (17)	0.71 (12)	0.12 (2)	0.06 (1)	0.18 (3)	0.00 (0)
verbal explanation (6)	0.50 (3)	0.50 (3)	0.00 (0)	0.00 (0)	0.00 (0)

Finally, the sample size and the number of observation hours were relatively small so our analysis is limited and focused on descriptive statistics. Here we briefly examine intracultural diversity and a few correlational relationships. First, a significant relationship existed between the frequency an infant experienced natural pedagogy and the frequency they imitated (*R*^2^=0.47, *p*=0.029). This relationship did not exist for demonstration or the total number of teaching episodes that an infant experienced. This suggests that natural pedagogy plays an important role in imitation. Infants did observe adults or children make a fire or use a knife and imitate them on their own, but the majority (about 70%, see table 4) of the cases of imitation occurred in the context of natural pedagogy or another type of teaching.

The frequency an infant was held in the caregiver's lap facing out (‘distributed teaching’) was not related to number of teaching events or episodes that an infant experienced. In other words, being in the lap facing out from a caregiver did not diminish the frequency of teaching.

It was interesting to find that the frequency an infant engaged in practice play was significantly related to the total number of teaching episodes she received (*R*^2^=0.64, *p*=0.006), but not to the frequency of natural pedagogy and only slightly to the frequency an infant received demonstrations (*R*^2^=0.41, *p*=0.047). No relationship existed between the frequency of teaching episodes, natural pedagogy or demonstration with general play. There was also no relationship between the two types of play.

Finally, we wanted to try and understand the frequent caregiver pointing and found significant relationships between frequency of caregiver pointing and number of teaching episodes (*R*^2^=0.51, *p*=0.021) and the frequency of practice play (*R*^2^=0.66, *p*=0.005), but no relationship with the frequency of imitation. We are not sure what to make of this since we do not know what the caregivers were pointing to, but it may be associated with the presumed functions of distribution teaching; caregivers, often parents, are pointing to other people and objects in and around the camp that children can learn from. Carer pointing without any clear association to teaching occurred about 10 times an hour on average, which was almost as frequent as the total number of teaching episodes per hour that infants received. In most cases, it appeared the caregivers were trying to draw the attention of the infant, but as mentioned above, it was difficult to determine given the infant focus of the camera. In other contexts (e.g. natural pedagogy), pointing is an important component of infant teaching and learning.

## 4. Summary and discussion

### 4.1 Summary

(i) The study identifies several forms of teaching that regularly occur during Aka hunter–gatherer infancy. Natural pedagogy, demonstration, task assignment, positive and negative feedback, and opportunity scaffolding were all observed on a relatively regular basis. Teaching episodes were usually brief and subtle, often lasting a few seconds. Episodes may be brief because they are low cost, because teachers tried to restrain their impact on the autonomy of the infant (see below), or because the teachers have intimate knowledge of the learners and do not need to modify their behaviour much to impact the learning process. Further research is needed to address these issues.
(ii) This was the first study to examine the natural pedagogy hypothesis of Gergely & Csibra [24] in a small-scale culture. Natural pedagogy occurred regularly among the Aka and was associated with the transmission of a broad range of skills and knowledge. As mentioned, we did not systematically evaluate the skill the infants acquired but skills transmitted by natural pedagogy were imitated by infants immediately after the event 41% of the time. But natural pedagogy was impacted by the Aka cultural context and interactions relied more on touch, physical proximity and pointing, and less on verbal exchange and motherese, which were common in Western laboratory-based samples [27]. Verbal explanations were rare and *no* instances of motherese were observed.
(iii) Aka infants observed and imitated skills and behaviours they naturally observed in the community, but they were more likely to do so in the context of natural pedagogy or demonstration where caregivers briefly drew their attention to particular objects or events. This questions generalizations from social–cultural anthropologists that observation and imitation, without any type of teaching, are the primary modes of learning.
(iv) Data were generally consistent with the hypothesis that teachers are likely to be biologically related to the learners [32]. Parents, especially mothers, were frequently the teacher. They were particularly active in the transmission of subsistence skills and knowledge, but less so with cultural norms where many individuals modified their behaviour to help infants learn. The data also question mathematical models that suggest teaching occurs only with a limited number of complex skills [33] and knowledge that cannot be acquired by observation and imitation alone. Many of the skills are easy to observe in the camp (see below) and various forms of teaching were used to engage infants in a range of relatively basic skills and knowledge.
(v) Some evidence existed for cooperative breeding [38], i.e. the investment in and concern for infants by individuals other than mother, in some forms of infant teaching. For instance, infants received negative feedback from many others, fathers and several others provided teaching episodes, caregivers frequently placed infants in their lap so they faced the entire group, and others often drew the attention of the infant to others in camp by pointing to them. The last two items, facing infants towards the group and pointing to others, potentially provide learning or investment opportunities from others and neither behaviour exists in great apes.
(vi) Limited support also exists for the Shennan & Steele [32] hypothesis that teaching by parents is likely to occur as early in the child's life as possible. Their model emphasizes parental transmission of skills, especially craft skills, during childhood and does not address teaching in infancy, but our data suggest that parents may start to teach as early as infancy to begin to provide age-appropriate learning opportunities to develop sensory–motor–cognitive foundations for the transmission and acquisition of complex skills later in life.
(vii) Various types and combinations of teaching exist, e.g. natural pedagogy with demonstration, negative feedback with redirect, task assignment with natural pedagogy, demonstration with body movement, etc. A taxonomy of teaching is necessary in order to evaluate the range, variability and contexts of different forms of teaching. It would be useful to build upon the taxonomy proposed Strauss & Ziv [25] for WEIRD cultures and a functionally based one suggested by Klein [2].
(viii) Teaching is used to transmit a broad range of skills and knowledge and the type of teaching varies with what is being transmitted and acquired. Natural pedagogy and demonstration were most likely to be used to transmit subsistence skills and knowledge, negative feedback and redirect were commonly used to promote the learning of cultural norms, while positive feedback was often associated with the transmission of how to dance and sing.

### 4.2 Discussion

The frequency of teaching in Aka infancy was unexpected. The first author observed parents teaching infants how to use axes and digging sticks while on net hunts 40 years ago but he had no idea it occurred this frequently. As mentioned, the setting (adult leisure time in camp) may have contributed to the high frequency, and more research is needed for longer time periods in diverse settings. Social–cultural anthropologists [15], cultural psychologists [17] and some evolutionary biologists [39] indicate that teaching is rare or non-existent in small-scale cultures. Social–cultural anthropologists and cultural psychologists seldom mention teaching, because they often use definitions and conceptions of teaching from formal education and their research problems frequently focus on identifying differences in learning between small-scale and WEIRD cultures. In addition to definition issues, focal and scan observational methods used by anthropologists may limit their ability to document teaching. The use of videotapes and microanalysis that focused on teaching within a narrow age range in infancy (months) enabled us to capture events that often lasted less than 3 or 4 s. Focal follow observational techniques that do not focus on teaching and record multiple events every minute and include children from a broad age range may miss these events. Whiten *et al.* [39] indicate teaching is rare or does not exist in hunter–gatherers, but the two citations used to support the statement were based upon reports from anthropologists whose research did not focus on teaching, did not use videotapes, and interviewed or conducted observations of a broad range of children.

Teaching, as defined in this paper, appears to be a regular component of hunter–gatherer learning. It occurs regularly in Aka infancy and a systematic examination of the cross-cultural literature on social learning in hunter–gatherers indicates teaching is a common process of learning across a wide range of skills and knowledge [19]. Current research [40] indicates that the frequency of teaching declines substantially with age (to two or three episodes per day) and that the age of the child influences who teaches: parents (vertical) in infancy and early childhood, older peers (horizontal) in middle childhood and other adults (oblique) in adolescence [41].

While teaching is a part of the hunter–gatherer children's learning experience and probably contributes to high fidelity transmission of skills, other processes such as observation, participation and practice, often emphasized by social–cultural anthropologists, are at least as important in the acquisition and refinement (fidelity) of essential skills. In this limited study, infants, on average, received 3.8 min of teaching during the 60 min of taping, but they practised the skills, often in a play context, for 9.3 min. Our current work with Chabu hunter–gatherers of Ethiopia indicates that indigenous views about how children learn are similar to the processes emphasized by social–cultural anthropologists; i.e. watching, listening, doing and participating are especially important in Chabu learning models. Advice and guidance (i.e. teaching) are part of Chabu models of learning, but they are not as salient to them as the other processes.

Natural pedagogy exists among the Aka but it is different from how it is described by researchers and experienced by infants in WEIRD cultures. We have described the frequent physical contact and general lack of verbal explanations or use of motherese during teaching episodes. Aka teaching episodes also regularly involved infants learning how to use sharp knives, machetes and digging sticks, tools that are often considered dangerous for infants in WEIRD cultures. Everyone in camp uses these tools, adults rarely limit their use by infants or children (adults gave these tools to infants in several episodes), and infants have plenty of opportunities to observe others using these tools.

From a Western perspective, parents also seemed to restrain themselves to minimize their teaching intervention with infants. For instance, in one episode an infant was cutting food rapidly with a knife as a parent sitting next to her watched, but the parent intervened for only a few seconds to adjust the infant's arm and never said anything during the episode. Aka and several other hunter–gatherer groups do not have a term for ‘teaching’, but among the Aka they use the word *mateya* to refer to advice or guidance. The term implies the child has the option whether or not to pay attention to the advice. Anthropologists working with hunter–gatherers emphasize the importance of respecting the autonomy of the child, a core value among most hunter–gatherers, and the general lack of teaching [42]. Among the Inuit, ‘teaching, scolding, or forcing teenagers to do something is considered discourteous because they do have reason, albeit under-developed, and thus must be accorded respect for their autonomy.’ [43]. The cultural values may help to explain why Aka teaching episodes during infancy were short and limited.

We agree with Kline [2] that it is problematic to talk about ‘teaching’ in general, that many different types of teaching exist in humans, and that the different types may have different functions. Several of the types of teaching identified in Kline (e.g. stimulus enhancement, evaluative feedback, direct teaching) emerged in in this study (table 3) but it is not clear nor do we have the space here to discuss the potential functions of the various forms of teaching in Aka infancy.

Strauss *et al.* [44] indicate most studies of teaching focus on learners rather than teachers. This study responded to this critique by focusing on teachers, but we need studies with small-scale cultures that demonstrate the cooperative nature of teaching and integrate teacher and learner perspectives, such as that conducted by Calero *et al.* [45]. We also need systematic studies on hunter–gatherer children's developmental abilities to teach. Some of the tapes in this study showed 2- and 3-year-old children using natural pedagogy with the focal infants. More extensive research is needed to evaluate the developmental sequence outlined by Strauss & Ziv [25].

### 4.3 Final comments

Teaching can enhance efficient high fidelity learning, but it can have a down side. Research by Bonawitz *et al.* [46] shows that teaching can limit what is learned. Their laboratory studies examined children's learning and exploration of toys with opaque functions. When a teacher provided a demonstration of one function of a toy that had multiple functions, the children focused almost exclusively on that function and did not explore other functions. Children that did not receive a demonstration were more likely to explore and discover multiple functions of the toy. This may be instructive, in particular, in a hunter–gatherer context where flexibility and autonomy are central to rapid adaptation to a mobile way of life, and may help to explain why teaching is rare after infancy and early childhood.

Overall, it is necessary to develop a truly integrated, multidisciplinary and holistic approach to understanding teaching. As Strauss & Ziv point out [25], considerable attention has been given to learning, but we know relatively little about teaching. Current research has focused on the cognitive and fitness enhancing dimensions of teaching, but little is known about how cultural beliefs and institutions influence the nature, frequency and effectiveness of teaching.

Years ago Harry Wolcott [47], p. 88 said the following about learning: ‘…what a fresh perspective anthropology might bring in rekindling interest in learning as a natural process rather than as an activity restricted to laboratories or schools’. In our opinion, the same could be said about teaching; it should not be restricted to schools and laboratories and our understanding of teaching will be enhanced with more extensive comparative ethnographic data.

## Data accessibility

The informed consent procedure did not include a statement about making the videotapes available to the public.
